# Mixed coronary plaque phantom analysis by photon-counting CT: impact of calcium and iodine on low-attenuation plaque detection

**DOI:** 10.1093/ehjimp/qyag119

**Published:** 2026-07-22

**Authors:** Bálint Szilveszter, Márton Kolossváry, Anikó Kubovje, Hugo Marques, Borbála Vattay, Zsófia Jokkel, Barbara Sipos, Milán Vecsey-Nagy, Melinda Boussoussou, Pál Maurovich-Horvat, Shawn Newlander, George Wesbey, Elliot McVeigh

**Affiliations:** Heart and Vascular Center, Semmelweis University, Városmajor Street 68, Budapest 1122, Hungary; Gottsegen National Cardiovascular Center, Budapest, Hungary; Physiological Controls Research Center, University Research and Innovation Center, Óbuda University, Budapest, Hungary; Heart and Vascular Center, Semmelweis University, Városmajor Street 68, Budapest 1122, Hungary; Unit of Cardiovascular Imaging (UNICA), Hospital da Luz, Lisbon, Portugal; Heart and Vascular Center, Semmelweis University, Városmajor Street 68, Budapest 1122, Hungary; Heart and Vascular Center, Semmelweis University, Városmajor Street 68, Budapest 1122, Hungary; Heart and Vascular Center, Semmelweis University, Városmajor Street 68, Budapest 1122, Hungary; Heart and Vascular Center, Semmelweis University, Városmajor Street 68, Budapest 1122, Hungary; Heart and Vascular Center, Semmelweis University, Városmajor Street 68, Budapest 1122, Hungary; Medical Imaging Centre, Semmelweis University, Budapest, Hungary; Scripps Clinic, Scripps Health, La Jolla, CA, USA; Scripps Clinic, Scripps Health, La Jolla, CA, USA; Departments of Medicine, Radiology, and Bioengineering, University of California San Diego, San Diego, CA, USA

**Keywords:** photon-counting detector CT, coronary CT angiography, low-attenuation plaque, virtual monoenergetic imaging, phantom study

## Abstract

**Aims:**

To test whether photon-counting detector CT (PCD-CT) improves low-attenuation plaque (LAP) detection compared with energy-integrating detector CT (EID-CT) using a mixed-plaque phantom and full-rotation helical images as high-resolution reference.

**Methods and results:**

We imaged a custom phantom housing three different sized mixed-plaque inserts (LAP_5.5mm_, LAP_4mm_, and LAP_7mm_) combining iodinated lumen (600/1000 HU), calcified arcs (200/800 mg/cc), and a narrow LAP central strip (1–1.5 mm, 75 HU), embedded in a fat-equivalent ring (−60 HU). Polychromatic and virtual monoenergetic images (40/70/100/130 keV) were reconstructed with soft (Qr40) and sharp (Qr72) kernels and quantum iterative reconstruction (QIR) levels. Findings were compared with a clinically used EID-CT protocol. HU line profiles and edge-width-at-half-maximum (EWHM) at the iodine–phantom interface were measured.

Reference EWHM was 0.125 mm. Cardiac half-rotation Qr40 reconstructions yielded EWHM 0.68–0.75 mm, whereas Qr72 approached reference values (0.18–0.23 mm). In LAP_5.5mm_, the 1-mm LAP layer was detectable on standard and UHR images with sharp kernels and higher QIR, with best attenuation fidelity using Qr72. For the smallest LAP_4mm_, only Qr72/QIR2 resolved the 1-mm LAP component, although measured attenuation remained elevated because of blooming from adjacent iodine and calcium. In LAP_7mm_, Qr72/QIR4 most closely reproduced the nominal LAP attenuation. Lower-keV images (40–70) increased LAP masking from iodine. EID-CT did not clearly depict LAP_4mm_.

**Conclusion:**

PCD-CT provides superior spatial resolution and HU fidelity for detecting LAP, particularly when using UHR images combined with sharp kernels (Qr72) and higher iterative strength (QIR 2–4). LAP detection improved at 130 keV vs. lower energies.

## Graphical Abstract

**Graphical Abstract:**
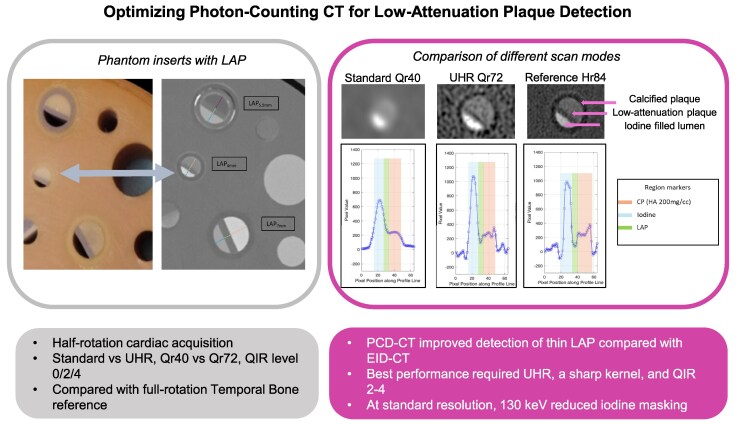

## Introduction

Low-attenuation plaque (LAP) identified on coronary computed tomography angiography (CCTA) has emerged as a potential marker of adverse cardiovascular events.^1–3^ Despite recent advances in scanner technology and image post-processing, several challenges persist in the quantitative assessment of LAP.^4–6^ Major concerns include the inter-reader, inter-scanner, and inter-platform reproducibility.^7,8^ A recent Society of Cardiovascular Computed Tomography expert consensus statement analysed the relative performance of quantitative coronary CT parameters and concluded that the accuracy of LAP quantification is poor and is strongly influenced by tube voltage, reconstruction settings, and intraluminal iodine concentration.^9^ Previous studies have shown that intraluminal iodine concentration has a major impact on LAP assessment, and published outcome studies have used different Hounsfield Unit (HU) thresholds to define LAP.^6,10,11^ In addition, lipid-rich and fibrous plaque components have significant HU overlap due to partial volume effects and tissue heterogeneity, which further limits reproducibility.

Photon-counting detector CT (PCD-CT) enhances image sharpness and lowers noise—by converting photons directly to electrical signals and using energy thresholds to reject electronic noise—thereby possibly improving LAP measurements.^12^ Cardiac ultra-high resolution (UHR) mode (120 × 0.2 mm collimation) improves spatial resolution and sharpens in-plane detail for more precise coronary assessment.^5,13^ Since its introduction in 2021, investigations have consistently shown improved image quality, better stenosis detection,^14^ lower rates of invasive angiography due to improved diagnostic accuracy^15^ and also cost effectiveness^16^ as compared with energy-integrating detector CT (EID-CT) for cardiac scans. One of the most important features of PCD-CT for plaque assessment is its ability to reduce blooming from high-density structures influencing the intra-plaque HU values within the adjacent LAP.^17,18^ However, there is still limited controlled data on how iodine and calcium affect LAP detection and whether different reconstruction kernel settings or energy levels with PCD-CT could overcome these limitations. Also, the impact of different reconstruction kernels on LAP detection has not been systematically evaluated against a high-resolution reference in a controlled phantom-based model using varying plaque dimensions.

Therefore, the primary aim of this study was to evaluate the detectability and attenuation fidelity of LAP-mimicking components in a mixed coronary plaque phantom using PCD-CT, focusing on the effects of adjacent iodine and calcium, reconstruction kernel, scan mode, and virtual monoenergetic level (VMI), with a high-resolution full-rotation helical temporal bone acquisition serving as reference. The secondary aim was to provide a pragmatic comparison with one clinically used EID-CT cardiac protocol.

## Methods

### Phantom design

**Figure 1:**
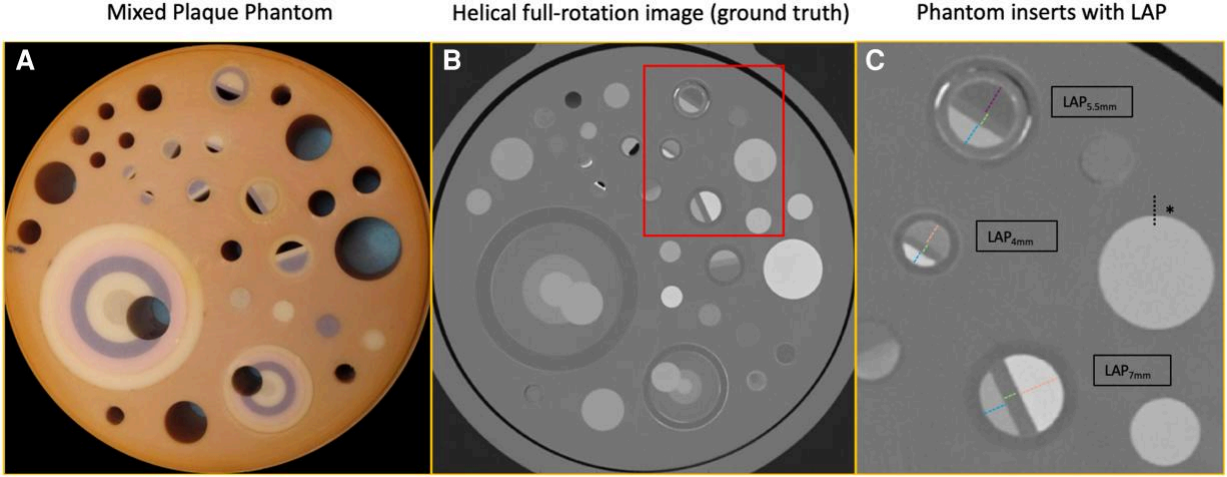
Mixed-plaque phantom for LAP detection using PCD-CT. Custom-made plaque phantom (*A*) with mixed plaque components to simulate the effects of different materials and scanning parameters on plaque assessment. Iodine based contrast media was added to attain the required concentration in the holes. EWHM was derived at the interface of the contrast-filled lumen and the phantom material (denoted by an asterisk), using a vertical edge profile across this region. The reference image shown in Figure (*B*) was obtained with a full-rotation, Temporal Bone imaging protocol. Phantom inserts (from top to bottom) measured 5.5, 4, and 7 mm in overall diameter (*C*): **Top:** LAP_5.5mm_: layers of a 2 mm iodine-filled lumen (diluted Iomeprol 400 mg I/mL, ∼600 HU on 120 kVp), 1 mm NCP_75HU_ and 2.5 mm NCP_-60HU_. **Middle:** LAP_4mm_: layers of a 1 mm iodine-filled lumen (diluted Iomeprol 400 mg I/mL, ∼1 000 HU on 120 kVp), a 1 mm LAP (75 HU), and a 2 mm calcified plaque (200 mg/cc), all encased in a −60 HU fat-equivalent ring. **Bottom:** LAP_7mm_: layers of a 2 mm high-density calcified plaque (800 mg/cc), 1.5 mm LAP (75 HU) and a 3.5 mm iodine-filled lumen (diluted Iomeprol 400 mg I/mL, ∼600 HU on 120 kVp), likewise surrounded by a −60 HU ring.

We imaged a custom-made mixed plaque phantom with three inserts representing coronary plaques of different sizes and compositions (*Figure 1*). The feature dimensions (from bottom left through top right profiles) were as follows:

1. LAP_5.5mm_: 5.5 mm total diameter; 2 mm iodine-filled arc lumen (∼600 HU at 120 kVp), 1 mm non-calcified plaque centre strip (non-calcified plaque NCP_75HU_), and 2.5 mm non-calcified semi-circle (NCP_−60HU_).
2. LAP_4mm_: 4-mm total diameter; 1 mm iodine-filled arc lumen (∼1000 HU at 120 kVp), 1 mm LAP centre strip (75 HU at 120 kVp), and 2-mm calcified arc (200 mg/cc, ∼280 HU at 120 kVp).
3. LAP_7mm_: 7 mm total diameter; 2.0 mm calcified semi-circle (800 mg/cc, ∼600 HU at 120 kVp, 1.5 mm LAP centre strip (∼75 HU at 120 kVp), and 3.5 mm iodine-filled arc lumen (∼1000 HU at 120 kVp).

Each coronary plaque–mimicking insert was encased in a −60 HU fat-equivalent ring. The phantom was designed to fit within the QRM thorax phantom (QRM GmbH, Moehrendorf, Germany) and a 35-cm fat-equivalent attenuation ring. The phantom setup was positioned in the isocentre of a dual-source photon-counting CT scanner with cadmium-telluride detectors (NAEOTOM Alpha peak, software version VA50, Siemens Healthineers, Forchheim, Germany). This configuration allowed us to isolate how adjacent high-attenuation structures (iodine, calcium) influence LAP visibility within the smaller LAP_4mm_ and larger LAP_7mm_ features. The LAP_5.5mm_ insert contained two non-calcified components with different nominal attenuation values, representing lipid-rich and fibrotic plaque surrogates, and enabled assessment of discrimination between adjacent low-attenuation soft-tissue-like regions. Representative cross-sectional images of the analysed plaque inlays are provided in Supplementary data online, *Figure S1*.

### CT acquisition protocol and image reconstruction

We applied three different scan protocols with ECG-simulated heart rate of 60/min (without motion) to trigger the gated scans: 1) cardiac gated CCTA sequential, half-rotation, standard resolution, 2) cardiac gated CCTA sequential, half-rotation, UHR, and 3) helical, ungated full-rotation Temporal Bone protocol (*Table 1*). Cardiac acquisitions were reconstructed from half-rotation data. Z-collimation was 144 × 0.4 mm for standard and 120 × 0.2 mm for UHR; both protocols used a 250-ms rotation. The effective mAs was set to 100. All standard resolution images were reconstructed using a slice thickness of 0.4 mm, a quantum iterative reconstruction (QIR) level of 0, 2, and 4, a medium smooth kernel quantitative regular (Qr40), and a sharper kernel (Qr72). We also created VMI-s (energy levels 4 070 100 and 130 keV) using the sharper kernel setting (Qr72). UHR reconstructions were generated with a slice thickness of 0.2 mm, using the same QIR levels (0, 2, and 4) and both the softer and sharper kernels. Reference images were obtained with an additional helical scan mode with full rotation, 0.5 s rotation time, Hr84 kernel, and higher radiation exposure (Temporal Bone protocol, 140 kVp, 194 mAs). All profiles were generated from images that were upsampled to a resolution of 0.1 × 0.1 mm per pixel using bicubic interpolation, to enable precise spatial interpolation. Each plaque inlay was scanned three times, and the resulting datasets were averaged to improve signal-to-noise ratio when mapping the exact profiles through the images. Averaging was applied uniformly across all acquisitions and did not alter the intrinsic spatial resolution, or the relative differences observed between reconstruction settings.

**Table 1:** Imaging and reconstruction parameters for standard and ultra-high resolution (UHR) and temporal bone (high-resolution reference) PCD-CT

Parameter	Standard mode	UHR mode	High-resolution reference standard
	Cardiac, sequential, half-rotation	Cardiac, sequential, half-rotation	Temporal Bone, helical, full-rotation
*Tube voltage (kVp)*	120	120	140
*Kernel*	Qr40 & Qr72	Qr40 & Qr72	Hr 84
*Field of view (mm)*	102	102	100
*Single collimation width (mm)*	0.4	0.2	0.2
*Rotation time (s)*	0.25	0.25	0.5
*Matrix size*	512 × 512	512 × 512	768 × 768
*Pixel dimensions (mm × mm)*	0.20 × 0.20	0.20 × 0.20	0.13 × 0.13
*Computed tomography dose index volume (mGy)*	13.98	14.79	55.05
*Energy levels (keV)*	40,70,100,130, T3D	T3D	T3D
*Focal spot (mm)*	0.8/1.2	0.8/1.2	0.6/0.7

### EID-CT protocol

We also evaluated the same phantom setup on an EID-CT system (Siemens SOMATOM Force) and used a clinically established prospective sequential half-rotation cardiac protocol at 120 kVp with 200 mm FOV, Bv40 kernel, and ADMIRE 3. For a comparison not dependent on kernel, the PCD-CT standard-resolution data were also reconstructed with the Bv40 kernel (EID-analogous), QIR 2, 0.4-mm slices, and the same 200 mm FOV (see Supplementary data online, *Table S1*).

### Image analysis

Primary endpoints were detectability of the LAP-mimicking component and attenuation fidelity relative to nominal phantom values. Secondary endpoints were edge width half maximum (EWHM) at the iodine-phantom interface and contrast-to-noise ratio (CNR) between adjacent plaque components.

We generated and compared line profiles drawn through the plaque phantom for different scan modes and reconstructions to measure the signal contrast between different components and to assess fidelity of HU values. Visual assessment was performed by one experienced cardiac CT reader (12 years of experience, B.Sz.) using predefined criteria for LAP visibility. Because the phantom geometry was predefined and known to the reader, the visual analysis was not intended to represent blinded diagnostic interpretation, but rather a structured technical assessment of whether the expected LAP-mimicking region could be visually separated from adjacent iodine, calcium, or non-calcified plaque components. Visual findings were a separate structured technical observation. For comparison of relative attenuation behaviour across different VMIs, line profiles were normalized to the highest attenuation value within each plaque inlay.

In MATLAB (version R2022a), EWHM was calculated along a vertical profile at the interface between a contrast-filled lumen (620 HU) and the phantom background material (47 HU) and was compared with the high-resolution Temporal Bone reference (*Figure 1*). CNR was calculated as the absolute difference in mean CT attenuation values between two plaque components in ImageJ (LAP vs. calcium for LAP_4mm_ and LAP_7mm_, and NCP_75HU_ vs. NCP_-60HU_ for LAP_5.5mm_) using rotated rectangular ROIs within the central strip, and within neighbouring features. The signal difference was divided by background noise defined as the standard deviation of an ROI placed in the central strip (see Supplementary data online, *Table S2*). CNR was used as a descriptive measure of component separability under the tested conditions. A formal analysis of detectability as a function of noise level was not performed. The reported CNR values reflect relative component separability in the averaged datasets and were not intended to represent single-scan clinical detectability.

## Results

### High-resolution reference standard images

**Figure 2:**
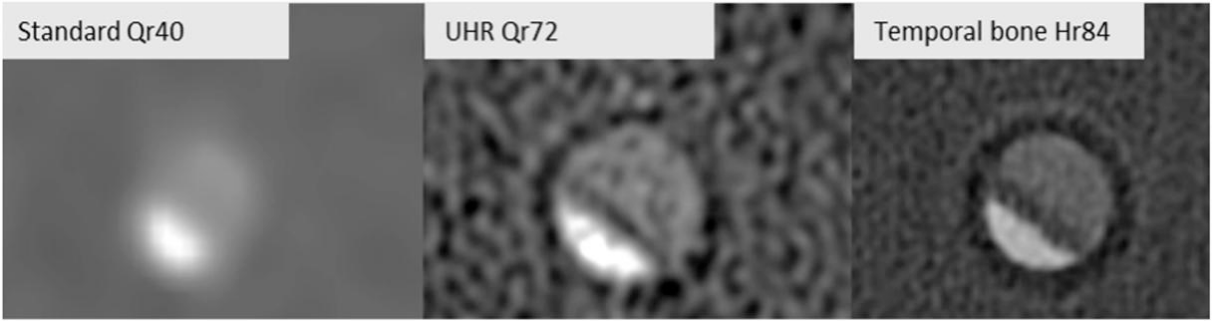
Low-attenuation plaque visualization on standard, UHR, and reference images using PCD-CT. Compared with the standard resolution (Qr40) and ultra-high resolution (Qr72) reconstructions, the Temporal Bone (Hr84) protocol provided markedly superior delineation of fine plaque morphology in the smallest plaque inlay (LAP_4mm_) using PCD-CT. This phantom design simulates a true-to-scale 4 mm coronary plaque, underscoring the relevance of these findings to real coronary artery dimensions.

**Figure 3:**
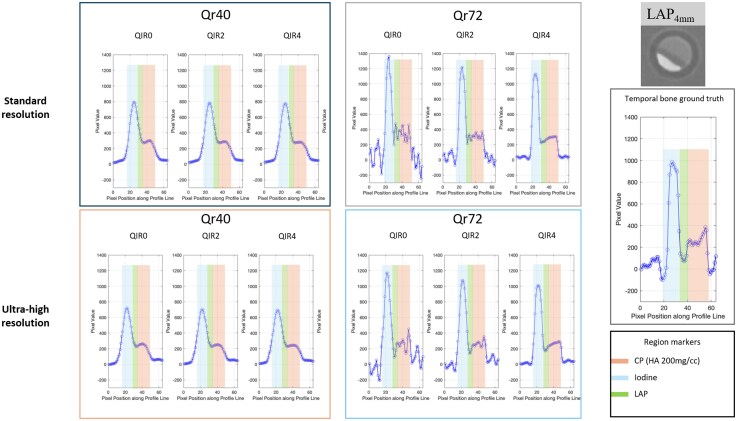
Line profile analysis of the mixed plaque inlay (LAP_4mm_) using standard resolution, UHR and reference images. Line profile comparison demonstrates sharper separation of different plaque components using the UHR mode with sharper kernels (Qr72) vs. standard scan mode with smoother kernels (Qr40). Each pixel in the profiles shown is 0.1 mm. Notably, using the sharper kernels (Qr72) with QIR2 or QIR4 provides the best visibility to detect the low-density ring (fat attenuation) and the LAP +75 HU component of the plaque. The local minimum corresponding to the LAP region remained higher than the reference value when using sharper kernels in UHR mode, due to blooming from adjacent iodine and calcified plaque. QIR 4 images achieved highest CNR values while preserving spatial fidelity. Region markers are color-coded for visualization: calcified plaque (CP) is shown in orange, iodine is shown in blue, and the low-attenuation plaque (LAP 75 HU) region is shown in green.

High spatial resolution scans using the Temporal Bone protocol (Hr84) on PCD-CT demonstrated clear visualization of all three plaques, with LAP consistently detectable. Even the smallest coronary plaque–like feature (LAP_4mm_) was clearly resolved for all of its components, demonstrating accurate edge delineation (*Figure 2*). The EWHM confirmed the excellent spatial resolution of this protocol (0.125 mm). Line profile analysis further showed that LAP was measured at ∼75 HU and the encapsulating fat ring at −60 HU, consistent with the true factory values for LAP_4mm_ (*Figure 3*). Similarly, HU fidelity was preserved for the two larger plaques, LAP_7 mm_ and LAP_5.5mm_.

### LAP detectability in the small mixed plaque (LAP_4mm_)

On visual inspection, only UHR mode with the Qr72 kernel (QIR0/QIR2) resolved the 1 mm LAP within the 4 mm plaque (LAP_4mm_); however, none of the smoother kernels provided clear delineation (*Figure 3*). Quantitative analysis confirmed that half-rotation cardiac acquisitions with smooth kernels had wide EWHM values ranging from 0.68–0.75 mm, whereas sharp kernels achieved values 0.18–0.23 mm, which are closer to those achieved by the Temporal Bone images.

The local minimum corresponding to the LAP region was detectable on UHR Qr72/QIR2, although its measured attenuation (∼170 HU) was higher than the reference value (75 HU), consistent with blooming from adjacent high-density components. (*Figure 3*).

### LAP detectability in the larger mixed plaque (LAP_7mm_)

**Figure 4:**
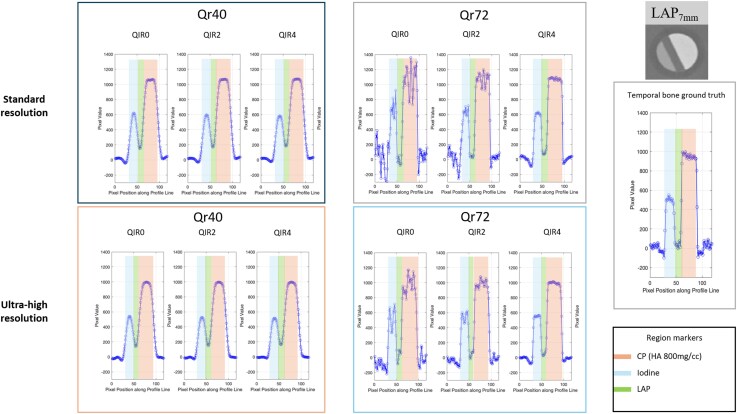
Line profile analysis of the larger mixed plaque inlay (LAP_7mm_) using standard resolution, UHR and reference images. Line profile comparison of the larger LAP_7mm_. The LAP-associated attenuation minimum was close to the reference value when using Qr72/QIR2 or Qr72/QIR4 for both the standard scan mode and UHR mode, indicating improved HU accuracy for assessing larger dimensions despite the higher calcium content and larger iodine lumen size. Each pixel in the line profiles represents 0.1 mm. Substantial increase in CNR is observed with QIR4, while visualization of plaque components and the corresponding HU values remain preserved (Qr72). Region markers are color-coded for visualization: calcified plaque (CP) is shown in orange, iodine is shown in blue, and the low-attenuation plaque (LAP 75 HU) region is shown in green.

In the larger mixed plaque (LAP7 mm), reconstructions with the sharp Qr72 kernel allowed clear visual identification of the 1.5-mm LAP central strip between the two high-density components across all QIR levels (*Figure 4*). Line profile analysis showed a distinct attenuation minimum corresponding to the LAP region, and HU fidelity was best preserved with Qr72/QIR4, yielding attenuation values close to the reference (∼75 HU) in both standard and UHR modes. In contrast, softer kernels failed to preserve HU accuracy and produced intra-plaque values around ∼200 HU. CNR values were high across several settings, consistent with the greater detectability of this larger insert, but were maximal with UHR Qr72/QIR4.

### Discrimination of adjacent non-calcified components (LAP_5.5mm_)

**Figure 5:**
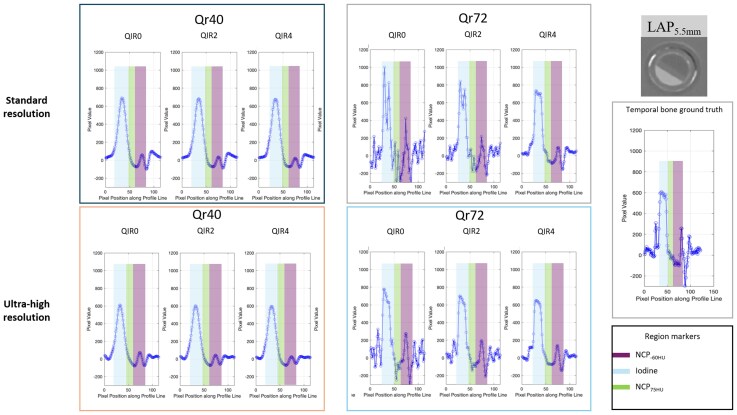
Line profile analysis of the mixed non-calcified plaque (LAP_5.5mm_) using standard resolution, UHR and reference images. Line profile comparison of the LAP_5.5mm_ with different NCP composition (75HU and −60HU). The NCP_75HU_ can be detected on Qr72/QIR4 and be distinguished from the iodine signal, although its value was higher (∼100 HU) than the high-resolution reference standard. The ultra-high resolution images provided slightly closer HU values of NCP_-60HU_ as compared with the standard resolution images. The reference images showed close to factory derived HU values for both NCP components. Colour coded regions of each plaque layer are shown on the lower right panel (Iodine was marked with blue, NCP_75HU_ central strip with green and the NCP_-60HU_ with purple). NCP: Non-calcified plaque; QIR: quantum iterative reconstruction; Qr: quantitative kernel.

The 1 mm wide NCP_75HU_ layer of the LAP_5.5mm_ plaque was readily detected in both standard and UHR reconstructions when sharp kernels were applied with the highest QIR level of 4. Attenuation fidelity was maintained with Qr72/QIR4, where the NCP_75HU_ and NCP_−60 HU_ layers were both reproduced in standard and UHR scans (*Figure 5*).

Across all three plaque types, either QIR 2 or QIR4 reconstructions provided the most favourable balance between edge definition and CNR (see Supplementary data online, *Table S2*). A full analysis of detectability vs. noise level was beyond the scope of this paper.

### Comparison with EID-CT for LAP detection

**Figure 6:**
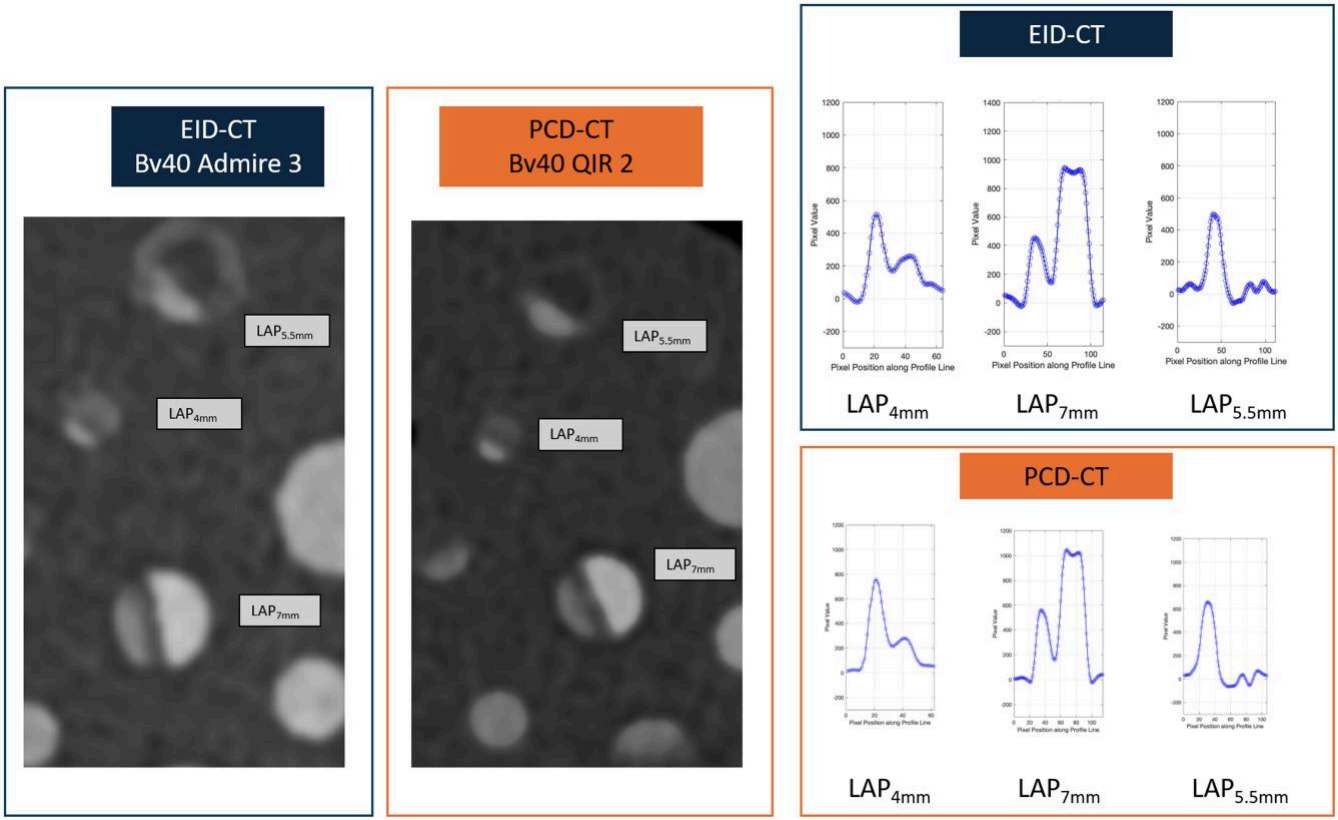
EID-CT vs. PCD-CT images for LAP assessment. Representative images of the three analysed plaques are shown, along with their corresponding line profiles acquired using two CT technologies: EID-CT (Bv40, 120 kVp) and PCD-CT (Bv40, QIR2, 120 kVp, T3D). Comparing the line profiles, the clinically used EID-CT protocol is unable to resolve the LAP layer and cannot differentiate NCP subcomponents with varying densities for LAP_5.5mm_. PCD-CT using protocols mimicking EID-CT settings offers a marginal improvement in LAP detection when using a soft kernel setting. To fully leverage the advantages of higher spatial resolution and improved CNR using PCD-CT, sharper kernel reconstructions or alternative VMIs are required. The present comparison does not represent the maximum achievable performance of EID-CT, and further optimization of EID-CT acquisition and reconstruction parameters may yield different results. Bv: Body vascular kernel; EID: energy-integrating detector; LAP: Low-attenuation plaque; PCD: photon-counting detector; QIR: quantum iterative reconstruction; Qr: quantitative kernel.

Using a clinical standard EID-CT protocol, the 1 mm LAP layer of the LAP_4mm_ and LAP_5.5mm_ could not be identified on visual inspection, whereas the larger 1.5-mm layer of LAP_7mm_ could be detected. Quantitative line profile analysis further confirmed the limited performance of EID-CT, as no distinct local minima corresponding to LAP were observed. Instead, the measured attenuation values were elevated relative to high-resolution reference standard, with ∼200 HU for LAP_4mm_, ∼170 HU for LAP_7mm_, and ∼100 HU for LAP_5.5mm_ (*Figure 6*). These findings demonstrate that, under standard clinical protocols, EID-CT failed to resolve LAP or differentiate non-calcified plaque subcomponents of varying density. An EID-like reconstruction generated from PCD-CT data using a comparable soft kernel and FOV provided only marginally improved visualization, further underscoring the necessity of sharp kernel settings for reliable LAP detection.

### Virtual monoenergetic images across different plaque inlays

Supplementary data online, *Figure S2* depicts the effects of different VMI-s on LAP detection (standard scan mode, Qr72/QIR4). Normalized line profile analysis across the 4, 7 mm, and 5.5 mm plaques demonstrated that lower energy VMI-s (40–70 keV) were limited by iodine blooming, resulting in unmeasurable local minima corresponding to LAP. At 100 keV, discrimination improved, whereas 130 keV provided the most favourable separation in all three inserts in this phantom configuration. In the LAP_4mm_, the LAP minimum was deepest and most sharply separated from adjacent calcified plaque at 130 keV. For the 5.5 mm plaque, only the 130 keV images reliably distinguished the 75 HU and −60 HU non-calcified components, with lower energies failing to resolve the two features (see Supplementary data online, *Figure S2*).

## Discussion

### Main findings

Iodine and calcified plaque substantially impair LAP detection and attenuation fidelity in half-rotation coronary CT imaging, particularly due to blooming artefacts and especially when evaluating small object diameters of interest. Full-rotation helical images served as a useful *in vitro* reference by accurately reproducing nominal HU values and providing excellent spatial resolution. PCD-CT imaging with sharp kernels (Qr72) achieved spatial resolution close to high-resolution reference standard, while softer kernels failed to delineate LAP, particularly in smaller plaques that better approximate true coronary dimensions. Using a clinically accepted EID-CT protocol resulted in poor discrimination of the LAP features, with limited ability to detect or quantify non-calcified plaque subcomponents. EID-like reconstructions from PCD-CT with soft kernels provided only marginal improvement, reinforcing the necessity of sharp kernels for LAP detection to maximize the benefits of PCD-CT. Furthermore, in this phantom configuration, selecting higher energy levels (130 keV) using spectral imaging improved LAP detection in the presence of high-density surroundings and also enhanced the differentiation between non-calcified plaque components.

### Current uncertainties in plaque quantification with PCD-CT

Determining the optimal reconstruction for plaque quantification is one of the major unresolved questions since the clinical implementation of PCD-CT. Although this technology is increasingly used for plaque analysis and may influence preventive and interventional decision-making, important limitations remain before broader clinical adoption. There are conflicting results on which protocol should be utilized for accurate and reproducible plaque quantification and characterization, and for serial CT scanning using both EID-CT and PCD-CT.^19^ Also, unified HU thresholds for LAP are not yet defined and validation studies against gold-standard IVUS remain limited.^20,21^ The phenomenon of ‘reverse blooming,’ whereby excessively high intraluminal iodine concentrations can mask LAP, further underscores the need for careful optimization of contrast and reconstruction protocols.^22^

### Rationale for controlled phantom analysis

Because previous studies have reported variability in optimal kernel and reconstruction settings, phantom investigations remain necessary to systematically evaluate the effects of kernel choice,^23^ scan mode, and energy levels in a controlled environment, particularly under the high intraluminal contrast conditions used in current clinical practice.^24–26^ In a prospective intra-individual study of 48 participants scanned with both EID-CT and PCD-CT within 30 days, Vecsey-Nagy *et al.* found that PCD-CT yielded significantly lower total plaque volumes but higher LAP volumes than EID-CT in the same individuals. In a complementary PCD-CT study using rod-shaped coronary stenosis phantoms, Skoog *et al.* showed that reconstruction kernel mainly affected LAP quantification, whereas slice thickness predominantly influenced calcified plaque volumes. They evaluated a set of kernels and 0.2- or 0.4-mm slice thickness and indicated that Bv64 outperformed Bv48 in terms of quantifying NCP volumes in both patients and a phantom with lower iodine attenuation. In contrast, our study addresses a more challenging configuration: detection of a 1-mm-thick, 75-HU plaque component positioned between 1-mm-thick 1000-HU iodine lumen attenuation and 2-mm-thick calcified plaque. Such a controlled phantom approach may help define technically robust reconstruction strategies before broader clinical implementation.

### Mechanistic interpretation of LAP detectability and attenuation fidelity

From a mechanistic and technical perspective, this controlled phantom framework helps explain how plaque size, adjacent high-attenuation structures, and reconstruction settings interact to influence LAP detectability and HU fidelity. The detection of LAP has become a central focus of recent research, as it represents a promising non-invasive approach for identifying coronary plaques at risk of rupture.^11,27,28^ At the same time, LAP is defined with varying HU cut-off values in the literature.^29,30^ Our prior in-vivo investigation using PCD-CT showed that different lower-energy VMIs (40–60 keV) photon energies substantially lower LAP volume using T3D as reference, underscoring the need for normalization protocols and standardized approaches to plaque quantification across studies and clinical practice.^13^

The phantom used in this study was designed with synthetic materials that mimic clinically relevant CT attenuation properties and was tailored to different challenging but realistic scenarios. A further strength of the model is that it enables comparison across plaques of different sizes and compositions, including one inlay containing two distinct NCP layers.

The preservation of HU fidelity is essential for accurate plaque characterization. HU values vary depending on the size of the measured object.^31,32^ In this controlled phantom framework, LAP detectability depended strongly on plaque size, spatial resolution, and reconstruction kernel. Only sharp reconstructions resolved the thinnest 1-mm LAP layer in the smallest insert, whereas the larger LAP_7mm_ remained visible across scan modes. When analysing the larger plaque (LAP_7mm_), the minimum attenuation values closely matched the reference images in both standard and UHR modes (T3D, Qr72/QIR4). In contrast, softer kernels led to poor detectability and failed to preserve HU accuracy. This suggests that commonly used half-rotation acquisitions with thicker slices and soft kernels are suboptimal for LAP quantification near adjacent high-density structures. The detector sampling frequency, and therefore, the spatial resolution, is reduced by 50% in cardiac CT compared with 360° helical full rotation Temporal Bone reference, which achieves the scanner’s maximal spatial resolution and likely explains its excellent agreement with factory-based CT attenuation values.^33^

Across the three plaque configurations, QIR4 generally yielded the highest CNR values, with UHR Qr72/QIR4 showing the strongest overall performance, particularly in the more challenging LAP_4mm_ and LAP_7mm_ inserts.

### Spectral imaging

By exploiting the spectral capabilities of current PCD-CT technology, high-resolution monoenergetic reconstructions can be generated to better delineate NCP. In our study, 130 keV improved LAP visualization in the presence of adjacent high-density structures and enhanced distinction between different NCP components (75 HU vs. −60 HU). For nearly a decade, VMIs generated with EID-CT have been known to reduce blooming artefacts, as first demonstrated using rapid kVp switching. While earlier-generation EID-CT scanners with VMI have demonstrated these benefits, the advent of newer-generation scanners, such as PCD-CT, provides an opportunity to further clarify the extent and reproducibility of artefact reduction and plaque characterization.^14,34–36^

### Comparison with EID-CT

When comparing currently used clinical protocols for EID-CT, we could not define the LAP_4mm_ and LAP_5.5mm_, which emulate the size of proximal coronary arteries.

Our findings should not be interpreted as questioning the clinical relevance of EID-CT-derived LAP. Instead, they highlight an important technical consideration: clinically used EID-CT protocols, particularly those reconstructed with soft kernels, may substantially underestimate the true extent of LAP and non-calcified plaque heterogeneity. However, this comparison should be interpreted with caution as the EID-CT protocol was a pragmatic reference representing a routine clinical protocol, rather than an exhaustive optimization of EID-CT. Therefore, the present comparison does not represent the maximum achievable performance of EID-CT, and further optimization of EID-CT acquisition and reconstruction parameters may yield different results. A recently published *in vivo* study suggests that UHR PCD-CT provides smaller voxels, sharper edge definition, and a wider dynamic range of attenuation values, allowing low-attenuation components to be separated more clearly from adjacent higher-attenuation structures. As a result, plaque voxels that were previously categorized as fibrotic tissue on EID-CT using soft kernels may be reclassified as LAP on PCD-CT with sharper kernel settings.^34^

### Possible clinical implications

The present results should be translated to *in vivo* coronary CTA with caution. The phantom provided a controlled environment in which plaque composition, geometry, iodine concentration, and calcium density were known, allowing systematic assessment of spatial resolution and attenuation fidelity. This is a major strength for technical validation, but it does not reproduce the full complexity of coronary imaging. In vivo coronary plaques are heterogeneous and affected by multiple factors including vessel curvature, cardiac motion, respiratory motion, and heart rate variability.

Clinically, improved LAP visualization may be relevant for risk stratification. More accurate separation of iodine, calcium, and low-attenuation components could improve estimation of LAP burden. This may also be important for serial plaque monitoring and for trials evaluating plaque-modifying therapies (mostly lipid-lowering and anti-inflammatory pharmacotherapy), where even small changes in LAP volume may be clinically meaningful.

Nevertheless, the phantom findings have important implications for clinical protocol development. They suggest that thin LAP components adjacent to iodine or calcium may be underestimated or missed when soft kernels or lower-energy reconstructions are used. Conversely, sharper PCD-CT reconstructions and higher-energy VMI may improve the technical conditions for LAP visualization. These observations should not be interpreted as proof of improved clinical risk prediction, but rather as a technical rationale for future *in vivo* validation studies comparing optimized PCD-CT protocols with invasive reference standards, histology where available, and clinical outcomes.

### Limitations

We acknowledge the limitations of our study. While there are more than 100 different combinations regarding imaging parameters (e.g. kV or mA selection) and reconstruction parameters (e.g. slice thickness, kernel, QIR) using the first-generation dual-source PCD-CT system, we carefully selected protocol parameters that align with routine clinical practice and reflect recent scientific advancements. Similarly, only a subset of VMIs was analysed, which is justified as it effectively highlights the key aspects of the results. The iodine lumen attenuation of 1000 HU at 120 kVp in this study is high (LAP_4mm_) and represents the upper tertile of clinical CCTA use.^37^ Lower iodine lumen attenuation could be expected to improve LAP detection (6). Our analysis was limited to the in-plane (*x–y*) dimensions of the plaque inlays. The *z*-axis was not evaluated, as the 10 mm longitudinal extent of the inlays minimized partial volume effects along this direction. This does not detract from the main conclusions, which are based on in-plane spatial resolution and HU fidelity. Visual assessment was performed by a single experienced reader and was not blinded to the expected phantom geometry. This limits the generalizability of the visual findings and may introduce expectation bias. However, the visual assessment was used as a very practical structured technical observation rather than as a diagnostic reader-performance assessment. In addition, we averaged images together to obtain accurate measurements of the profiles, we did not evaluate the detectability as a function of CNR. Noise level will vary substantially between patients; therefore, a full evaluation of detectability would include changing the noise level and using an optimal detector.

### Conclusion

PCD-CT provides superior spatial resolution and HU fidelity for identifying LAP and lipid-rich plaque components, particularly when using UHR images combined with sharp kernels and higher iterative strength. LAP detection improved at 130 keV vs. lower photon energies for standard reconstructions. Full-rotation helical scans can serve as a reference for further research as they reproduce reference attenuation values. EID-CT and soft reconstructions failed to resolve thin LAP and underestimated non-calcified plaque heterogeneity. These findings support technical optimization of coronary PCD-CT protocols and warrant further *in vivo* validation.

## Supplementary Material

qyag119_Supplementary_Data

## Data Availability

The datasets generated and analysed during the current study are available from the corresponding author on reasonable request.

## Lead author biography

Dr Bálint Szilveszter is an Assistant Professor of Cardiology at the Heart and Vascular Center, Semmelweis University, Budapest, Hungary, where he heads the Cardiac Computed Tomography Working Group and leads the Scientific Cardiac Computed Tomography Program. He is a cardiologist with doctoral training in cardiovascular imaging, and his research focuses on coronary computed tomography angiography, atherosclerotic plaque imaging and quantitative plaque assessment, spectral and photon counting computed tomography, computed tomography perfusion imaging, computed tomography–based fractional flow reserve, and secondary prevention in coronary artery disease. He has authored more than 100 peer–reviewed publications and has received national competitive research support and awards, multiple fellowships, and scholarships. He has completed international training at several European centers and at Massachusetts General Hospital and Harvard Medical School, and he is actively involved in teaching and mentorship as a clinical instructor in cardiac imaging and supervisor of doctoral and undergraduate researchers.
