# Supplementary material for: Mixed coronary plaque phantom analysis by photon-counting CT: impact of calcium and iodine on low-attenuation plaque detection
Source: Eur Heart J Imaging Methods Pract. 2026 Jul 22;4(3):qyag119. doi: 10.1093/ehjimp/qyag119 (PMC13390644; doi:10.1093/ehjimp/qyag119)
Supplement: qyag119_Supplementary_Data [file qyag119_supplementary_data.zip › Supplementary_legends.docx]

**Supplementary Material**

**Supplementary Figure S1.** Representative enlarged images of the analysed LAP inlays using standard and UHR reconstructions and different QIR levels.

QIR: quantum iterative reconstruction; Qr: quantitative kernel

Alt text: Enlarged images of the plaque inserts reconstructed with standard and ultra-high-resolution protocols at different iterative reconstruction levels, illustrating the effect of reconstruction settings on plaque component visibility.

**Supplementary Figure S2.** Normalized intensity profiles across the plaques at monoenergetic levels of 40, 70, 100, and 130 keV.

Normalized intensity profiles across the 4 mm, 7 mm, and 5.5 mm plaques reconstructed at 40, 70, 100, and 130 keV (Qr72, QIR4). Each plaque layer is marked with a bar above the profiles. At lower energies (40–70 keV), iodine blooming elevates the curves and obscures LAP and non-calcified plaque subcomponents (LAP regions could not be distinguished from the surrounding regions). On the normalized plots at 130 keV (purple line), the LAP is deepest and most sharply defined, providing optimal separation from calcified plaque in the 4 mm and 7 mm inserts and clear discrimination of the 75 HU and –60 HU components in the 5.5 mm plaque. Moreover, the -60 HU fat ring resembling peri-coronary adipose tissue shows substantial changes across different energy levels, underscoring how energy selection influences both soft-tissue and adipose contrast. Color-coded region markers are added for better visualization.

CP: Calcified plaque**;** LAP: Low-attenuation plaque**;** NCP: Non-calcified plaque

Alt text: Normalized line profiles of the three plaque inserts reconstructed at 40, 70, 100, and 130 keV, showing reduced iodine blooming and improved separation of low-attenuation plaque and non-calcified components at higher monoenergetic levels, especially 130 keV.

**Supplementary Table S1.** Image acquisition and reconstruction parameters for the comparison of PCD-CT with clinically used EID-CT protocol.

**Supplementary Table S2.** Relative Contrast-to-Noise Ratio (CNR) Values for Plaque 1–3 Across Different CT Acquisition Protocols

CNR: Contrast-to-Noise Ratio, QIR: Quantum Iterative reconstruction, UHR: Ultra-high resolution

CNR was defined as the difference between the central LAP strip and the neighbouring features: calcified component or, for the 5.5-mm inlay between NCP_75HU_ and NCP_-60HU_. Noise was estimated from the root-mean-square-deviation measured in the background.  Noise level will change with patient size, so these CNR values are only relative to each other for this evaluation.
