# Supplementary material for: Mixed coronary plaque phantom analysis by photon-counting CT: impact of calcium and iodine on low-attenuation plaque detection
Source: Eur Heart J Imaging Methods Pract. 2026 Jul 22;4(3):qyag119. doi: 10.1093/ehjimp/qyag119 (PMC13390644; doi:10.1093/ehjimp/qyag119)
Supplement: qyag119_Supplementary_Data [file qyag119_supplementary_data.zip › Table S1_ (1).docx]

**Supplementary Table S1.** Image acquisition and reconstruction parameters for the comparison of PCD-CT with clinically used EID-CT protocol.

| Parameter | PCD-CT | EID-CT |  |
| --- | --- | --- | --- |
|  | Cardiac, sequential, half-rotation, standard resolution | Cardiac, sequential, half-rotation | |
| **Tube voltage (kVp)** | 120 | 120 | |
| **Kernel** | Bv40 | Bv40 | |
| **Field of view (mm)** | 200 | 200 | |
| **Single collimation width (mm)** | 0.4 | 0.6 | |
| **Rotation time (s)** | 0.25 | 0.25 | |
| **Matrix size** | 512x512 | 512x512 | |
| **Pixel dimensions (mm x mm)** | 0.39x 0.39 | 0.39x 0.39 | |
| **Computed Tomography Dose Index volume (mGy)** | 13.98 | 11.70 | |
| **Energy levels (keV)** | 40,70,100,130, Polychromatic | Polychromatic | |
| **Focal spot (mm)** | 0.8/1.2 | 0.8 | |
