# Supplementary material for: Mixed coronary plaque phantom analysis by photon-counting CT: impact of calcium and iodine on low-attenuation plaque detection
Source: Eur Heart J Imaging Methods Pract. 2026 Jul 22;4(3):qyag119. doi: 10.1093/ehjimp/qyag119 (PMC13390644; doi:10.1093/ehjimp/qyag119)
Supplement: qyag119_Supplementary_Data [file qyag119_supplementary_data.zip › Table S2.docx]

**Supplementary Table S2.** Relative Contrast-to-Noise Ratio (CNR) Values for Plaque 1–3 Across Different CT Acquisition Protocols

| **Scan Mode** | **Kernel** |  | **LAP_4mm_** | **LAP_7mm_** | **LAP_5.5mm_** |
| --- | --- | --- | --- | --- | --- |
| Standard | Qr40 QIR0 |  | -1.06 | 9.86 | 4.53 |
| Standard | Qr40 QIR2 |  | 1.00 | 9.53 | 4.82 |
| Standard | Qr40 QIR4 |  | 0.22 | 15.02 | 5.01 |
| Standard | Qr72 QIR0 |  | 0.38 | 2.14 | 0.66 |
| Standard | Qr72 QIR2 |  | 0.51 | 4.36 | 1.33 |
| Standard | Qr72 QIR4 |  | 4.28 | 18.25 | 2.99 |
| UHR | Qr40 QIR0 |  | 1.99 | 13.59 | 3.88 |
| UHR | Qr40 QIR2 |  | 0.99 | 9.89 | 3.88 |
| UHR | Qr40 QIR4 |  | 0.17 | 22.94 | 3.87 |
| UHR | Qr72 QIR0 |  | 1.92 | 3.35 | 0.77 |
| UHR | Qr72 QIR2 |  | 2.50 | 7.90 | 1.78 |
| UHR | Qr72 QIR4 |  | 5.87 | 30.51 | 5.14 |

CNR: Contrast-to-Noise Ratio, QIR: Quantum Iterative reconstruction, UHR: Ultra-high resolution

CNR was defined as the difference between the central LAP strip and the neighboring features: calcified component or, for the 5.5-mm inlay between NCP_75HU_ and NCP_-60HU_. Noise was estimated from the root-mean-square-deviation measured in the background. Noise level will change with patient size, so these CNR values are only relative to each other for this evalutation.
