# Supplementary figures and images for: Mixed coronary plaque phantom analysis by photon-counting CT: impact of calcium and iodine on low-attenuation plaque detection
Source: Eur Heart J Imaging Methods Pract. 2026 Jul 22;4(3):qyag119. doi: 10.1093/ehjimp/qyag119 (PMC13390644; doi:10.1093/ehjimp/qyag119)

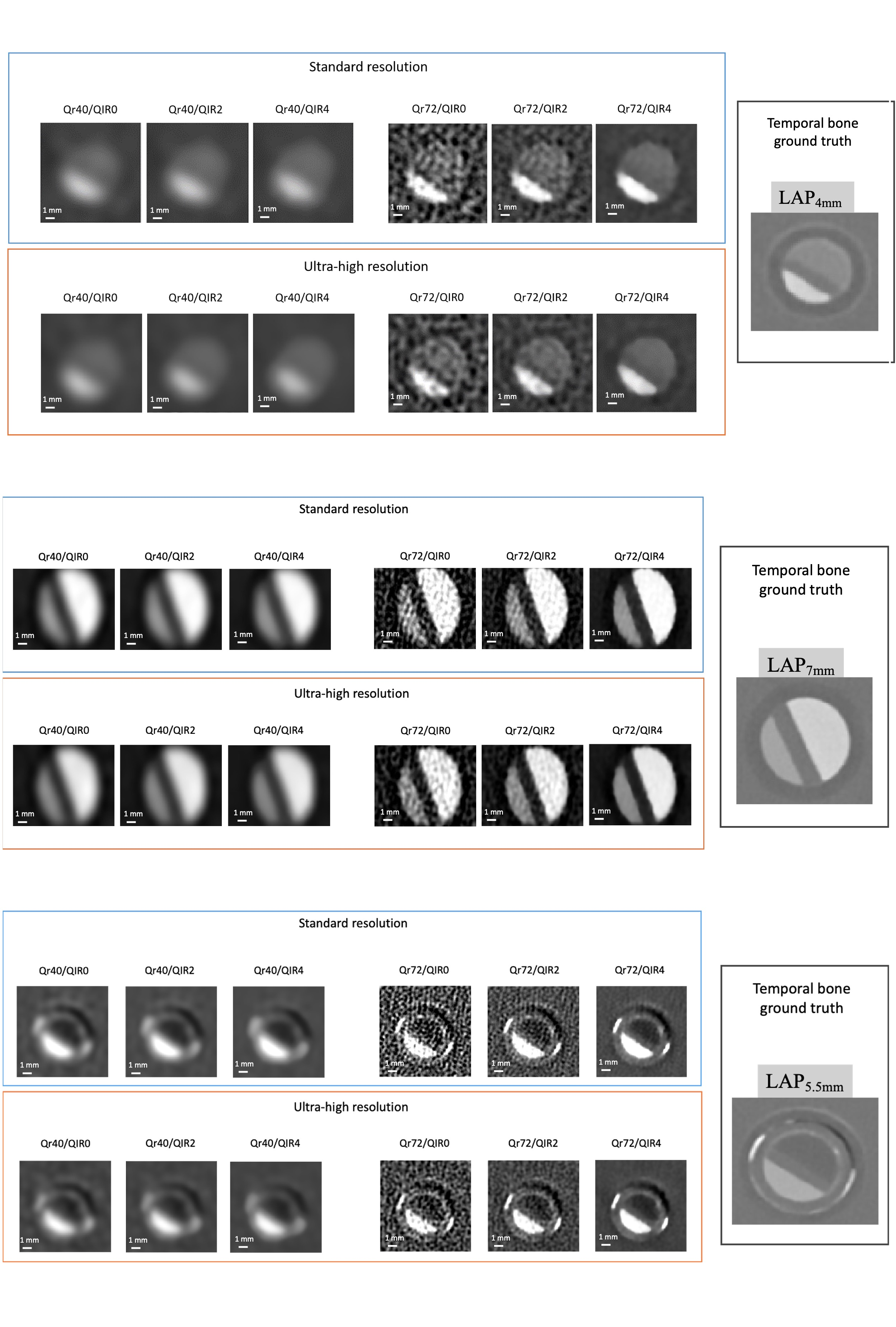

Supplement: qyag119_Supplementary_Data [file qyag119_supplementary_data.zip › Supplemental Fig 1.tif]

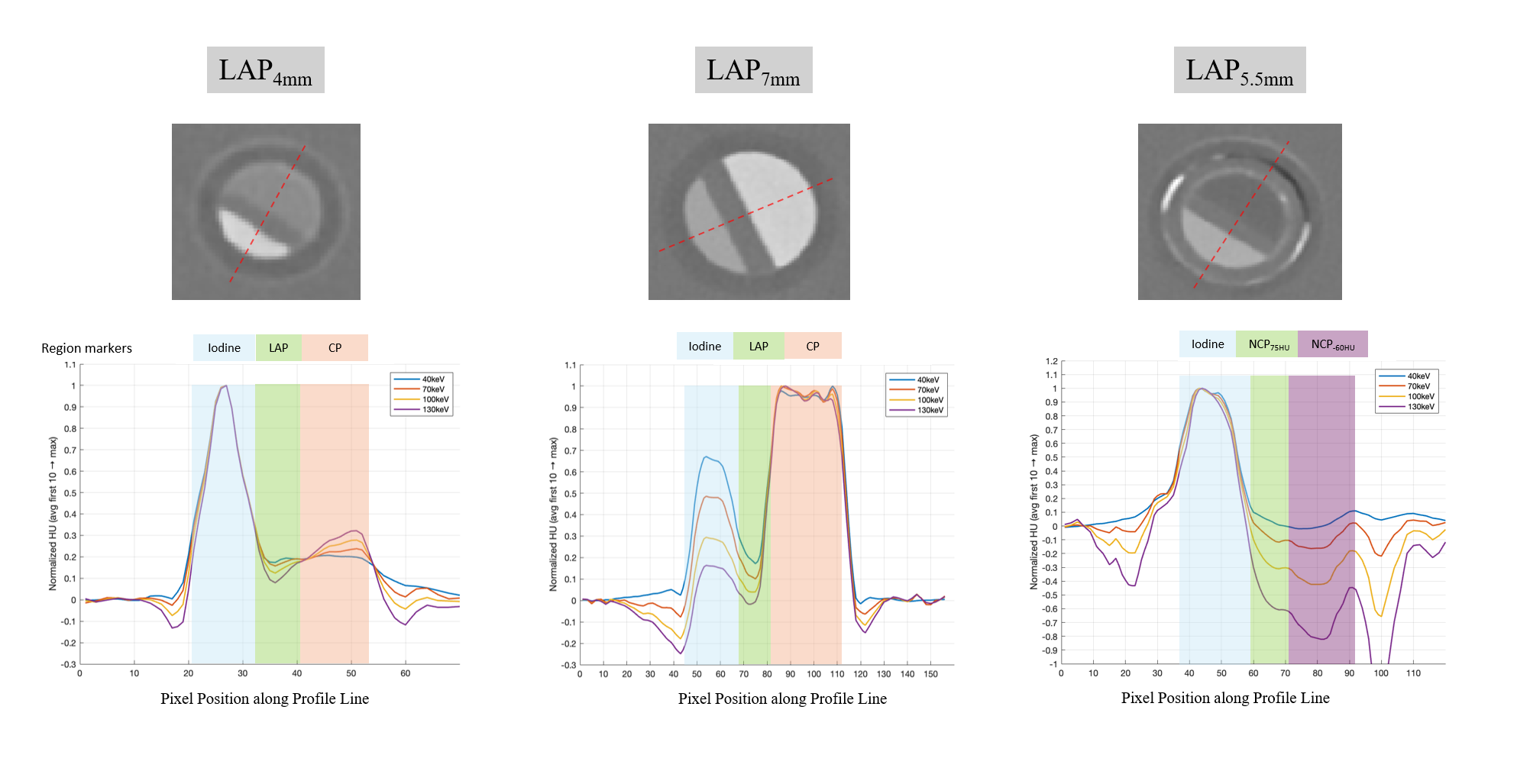

Supplement: qyag119_Supplementary_Data [file qyag119_supplementary_data.zip › Supplementary Fig 2.tif]
